# Long-term effect of medium cut-off dialyzer on middle uremic toxins and cell-free hemoglobin

**DOI:** 10.1371/journal.pone.0220448

**Published:** 2019-07-26

**Authors:** Nam-Jun Cho, Samel Park, Md Imtiazul Islam, Ho-Yeon Song, Eun Young Lee, Hyo-Wook Gil

**Affiliations:** 1 Department of Internal Medicine, Soonchunhyang University Cheonan Hospital, Cheonan, Korea; 2 Department of Microbiology and Immunology, School of Medicine, Soonchunhyang University, Cheonan, Korea; Istituto Di Ricerche Farmacologiche Mario Negri, ITALY

## Abstract

The medium cut-off (MCO) dialyzer has shown good clearance of large middle molecules, but its long-term effects are unclear. We investigated whether MCO hemodialysis (HD) over one year could reduce middle molecule levels and cell-free hemoglobin (CFH), without albumin loss. A prospective cohort study in 57 hemodialysis patients was conducted. The patients were assigned to the MCO dialyzer group or the high-flux dialyzer group, according to the HD machine they used. The reduction ratio (RR) and one-year changes in small and middle molecules and CFH were analyzed. Over a 12-month follow-up, MCO HD did not reduce the serum levels of middle molecules (lambda free light chain [FLC], from 135.7 ± 39.9 to 132.0 ± 39.1 mg/L; kappa FLC, from 168.2 ± 58.5 to 167.7 ± 65.8 mg/L; β2-microglobulin, from 25.6 ± 9.6 to 28.4 ± 4.8 mg/L) or albumin (from 3.96 ± 0.31 to 3.94 ± 0.37 g/dL). MCO HD provided excellent RR of lambda FLC (49.3 ± 10.3%), kappa FLC (69.6 ± 10.4%) and β2-microglobulin (80.9 ± 7.3%), compared to high-flux HD. CFH was also removed well during an MCO HD session (RR of CPH, 85.5 [78.7–97.3] %), but long-term change was not significant (from 57.8 [46.2–79.1] to 62.0 [54.6–116.7] mg/L). The MCO dialyzer can be used effectively and safely in conventional HD settings, but long-term effects on large middle molecules and CFH were not significant. Further studies are needed to verify clinical benefits of the MCO dialyzer.

## Introduction

When end-stage renal disease (ESRD) develops, various organ functions deteriorate due to accumulation of uremic toxins, leading to high mortality rates [1, 2]. Uremic toxins exhibit a broad array of physicochemical characteristics, mechanisms of generation, and pathobiological actions at the cellular and molecular levels [3]. According to the size and protein-binding properties of toxins, uremic toxins can be classified as small molecules (<500 Da), middle molecules (>500 Da–60 kDa), and protein-bound molecules [4].

Traditional hemodialysis (HD) membranes have focused on removal of small molecule toxins, such as urea and creatinine, but gradually middle molecule toxins have attracted attention because of their impact on disease progression and mortality [5, 6]. After introduction of high-flux membranes and on-line hemodiafiltration (ol-HDF), clearance of β2-microglobulin dramatically improved, but larger middle molecules, which also function as critical toxins, were still insufficiently removed [6–8]. Recently, medium cut-off (MCO) dialyzers, a novel class of membranes with a higher pore size designed to increase the removal of larger middle molecules were introduced into clinical settings [9]. Recent studies on the use of the MCO dialyzer in HD patients have shown efficient removal of β2-microglobulin and larger middle molecules, such as kappa and lambda free light chains (FLC), complement factor D, and α1-microglobulin [10–12]. However, because data to date were based on short-term use of the dialyzer, longer observational studies are essential to confirm the efficacy and safety of the MCO dialyzer.

Cell-free hemoglobin, a 62.6 kDa protein in plasma, can be generated from mechanically stressed erythrocytes during extracorporeal therapies [13, 14]. It is increased during HD sessions and is much higher in HD patients than in the normal population [15, 16]. The adverse effects of CFH were previously reported in studies of hemolytic anemia, malaria, sepsis, and blood transfusions [17–20]. Increased levels of CFH in HD can cause decline of nitric oxide bioactivity and subsequent endothelial dysfunction [15]. We were interested in CFH because a previous *in vitro* study revealed that some degree of CFH could be eliminated during HD with the MCO dialyzer [21].

Taken together, we investigated whether using the MCO dialyzer for up to 12 months could decrease the levels of middle molecules and CFH, while keeping serum albumin steady.

## Materials and methods

### Study design and patients

This prospective observational study was performed in patients with ESRD from the Dialysis Unit of Soonchunhyang University Cheonan Hospital in Cheonan, Republic of Korea. The study was conducted in accordance with the Declaration of Helsinki, and the protocol was approved by the Institutional Review Board of the Soonchunhyang University Cheonan Hospital (2018-07-033). All study participants provided informed written consent prior to study enrollment.

In the dialysis facility, three different kinds of dialysis machines, including Artis (Gambro Dasco, Medolla, Italy), AK200 ULTRA S (Gambro AB, Lund, Sweden), and Fresenius Medical Care (FMC) 5008 dialysis machines (FMC Deutschland, Bad Homburg, Germany) have been used. The kind of machine assigned to the patients was the machine available at the first HD in this facility, and was not changed thereafter. The membrane selected for use was either Gambro’s (Revaclear 400 or Theranova 400) or FMC’s (FX CorDiax 80), according to the particular dialysis machine used by the patient. Before October 2017, when the Theranova 400 dialyzer was introduced in the facility, all patients were on HD with a high-flux membrane (Revaclear 400 and FX CorDiax 80), then the Revaclear 400 membrane was changed to an MCO membrane (Theranova 400). Therefore, there has been a high-flux HD group (FX CorDiax 80) and an MCO HD group (Theranova 400) in the facility since October 2017.

Inclusion criteria included age over 20 years and ESRD requiring HD treatment thrice weekly for at least six months. Exclusion criteria were patients who were not clinically stable, had hemolytic disease, had history of blood transfusion during the study period or patients who declined to participate in the study. Patients were followed for a year, from October 2017 to September 2018. The dialysis regimen of each patient, including blood flow, dialysate flow, and treatment time duration per session was not altered. All patients were treated with bicarbonate dialysate of ultrapure quality, and dialyzers were used only once.

### Samplings and analyses

Baseline pre-dialysis blood samples were drawn before the introduction of the MCO membrane, and follow-up pre-dialysis and post-dialysis samples were drawn one year after baseline. Blood was collected in serum-separating tubes and allowed to stand for 30 minutes, then centrifuged for 10 minutes at 3,000 rpm at room temperature within one hour. The serum was extracted and stored at -70°C until analysis. Immuno-turbidimetric assays were used for measurements of kappa FLC, lambda FLC, and β2-microglobulin. An electrochemilumino-immunoassay (ECLIA) was used for measurement of vitamin B12. Cell free-hemoglobin in serum was measured using a hemoglobin colorimetric assay kit (Biovision, Milpitas, CA), according to the manufacturer’s instructions. Residual renal functions were calculated by the equations derived from the previous studies using the baseline β2-microglobulin levels [22, 23].

### Calculations

Using pre-dialysis and post-dialysis samples, the reduction ratio (RR) of small and middle molecules was calculated using the following formula:

$$RR(\%)=\left(1-\frac{C_{post}}{C_{pre}}\right)\times 100$$

where *C*_*pre*_ and *C*_*post*_ are measured serum concentrations of the solute before and at the end of the HD session, respectively. Post-dialysis concentrations of middle molecules were corrected for hemoconcentration using a single-compartment kinetic model with the following formula, according to Bergström and Whele [24]:

$$C_{post-corr}=\frac{C_{post}}{\left(1+\frac{BW_{pre}-BW_{post}}{0.2\times BW_{post}}\right)}$$

where *BW*_*pre*_ and *BW*_*post*_ are patient’s body weight before and at the end of the HD session, respectively.

### Statistical analyses

Statistical analyses were performed using R version 3.4.3 (The R Foundation for Statistical Computing, Vienna, Austria). Categorical variables were expressed as counts (percentage), normally distributed continuous variables as means ± SD, and non-normally distributed continuous variables as medians (interquartile ranges). Differences between two independent groups were analyzed by Student’s t-tests for normally distributed continuous variables, and by Mann–Whitney U test for non-normally distributed continuous variables. Differences between two dependent groups were analyzed by paired samples t-tests for normally distributed continuous variables, and by Wilcoxon signed-rank test for non-normally distributed continuous variables. Categorical variables were analyzed using the Pearson's Chi-squared test or Fisher’s exact test, as appropriate. The linear mixed model was used to examine the difference of the monthly doses of erythropoietin-stimulating agents (ESA) between high-flux and MCO groups. *P*-values < 0.05 were regarded as statistically significant, and two-tailed tests were performed in all hypothesis tests.

## Results

### Patient characteristics at baseline

A total of 57 participants were enrolled in this study, which included 19 high-flux HD patients and 38 MCO HD patients. Their mean age was 54.6 ± 10.7 years, with a dry weight of 58.0 ± 12.2 kg, and median dialysis vintage of 73 (range, 37–178) months. Baseline clinical characteristics according to dialyzer types are presented in Table 1. There was no significant difference between high-flux HD patients and MCO HD patients. There was no patient who used a temporary HD catheter, and 91% of patients had a native arteriovenous fistula as their vascular access.

**Table 1:** Baseline characteristics of patients enrolled in the study according to dialyzer types.

	High-flux HD (*n* = 19)	MCO HD (*n* = 38)	*P*-value
Age, years	56.4 ± 10.4	53.7 ± 10.9	0.378
Gender, *n* (%)			0.393
∙ Male	13 (68.4)	20 (52.6)	
∙ Female	6 (31.6)	18 (47.4)	
Dry weight, kg	58.3 ± 11.2	57.9 ± 12.8	0.891
Primary renal disease, *n* (%)			0.100
∙ Diabetic nephropathy	5 (26.3)	10 (26.3)	
∙ Hypertensive	6 (31.6)	11 (29.0)	
∙ Glomerulonephritis	4 (21.1)	16 (42.1)	
∙ Polycystic kidney disease	4 (21.1)	1 (2.6)	
Dialysis vintage, months	120.0 (28.0–192.5)	71.5 (45.0–151.3)	0.767
Session length, min	246.1 ± 12.3	242.2 ± 5.4	0.102
Vascular access, *n* (%)			1.000
∙ Native AV fistula	17 (89.5)	35 (92.1)	
∙ PTFE graft	2 (10.5)	3 (7.9)	
Blood flow, mL/min	289.5 ± 19.6	294.2 ± 18.1	0.365
Ultrafiltration volume, L	2173.7 ± 918.9	2652.6 ± 1236.0	0.141
Kt/V per session	1.85 ± 0.31	1.86 ± 0.34	0.993
Estimated RRF (Shafi et al.)	1.15 (0.64–1.49)	1.05 (0.67–1.69)	0.980
Estimated RRF (Vilar et al.)	1.46 (0.56–2.14)	1.61 (1.01–3.38)	0.461

Data are presented as mean ± SD, median (interquartile range), or count (%), as appropriate. *P*-values were calculated by Student’s t-test for normally distributed continuous variables, by Mann–Whitney U test for non-normally distributed continuous variables, and by Pearson’s Chi-squared test or Fisher’s exact test for categorical variables. MCO, medium cut-off; HD, hemodialysis; AV, arteriovenous; PTFE, polytetrafluoroethylene; RRF, residual renal function.

### Changes after one-year treatment

**Fig 1:**
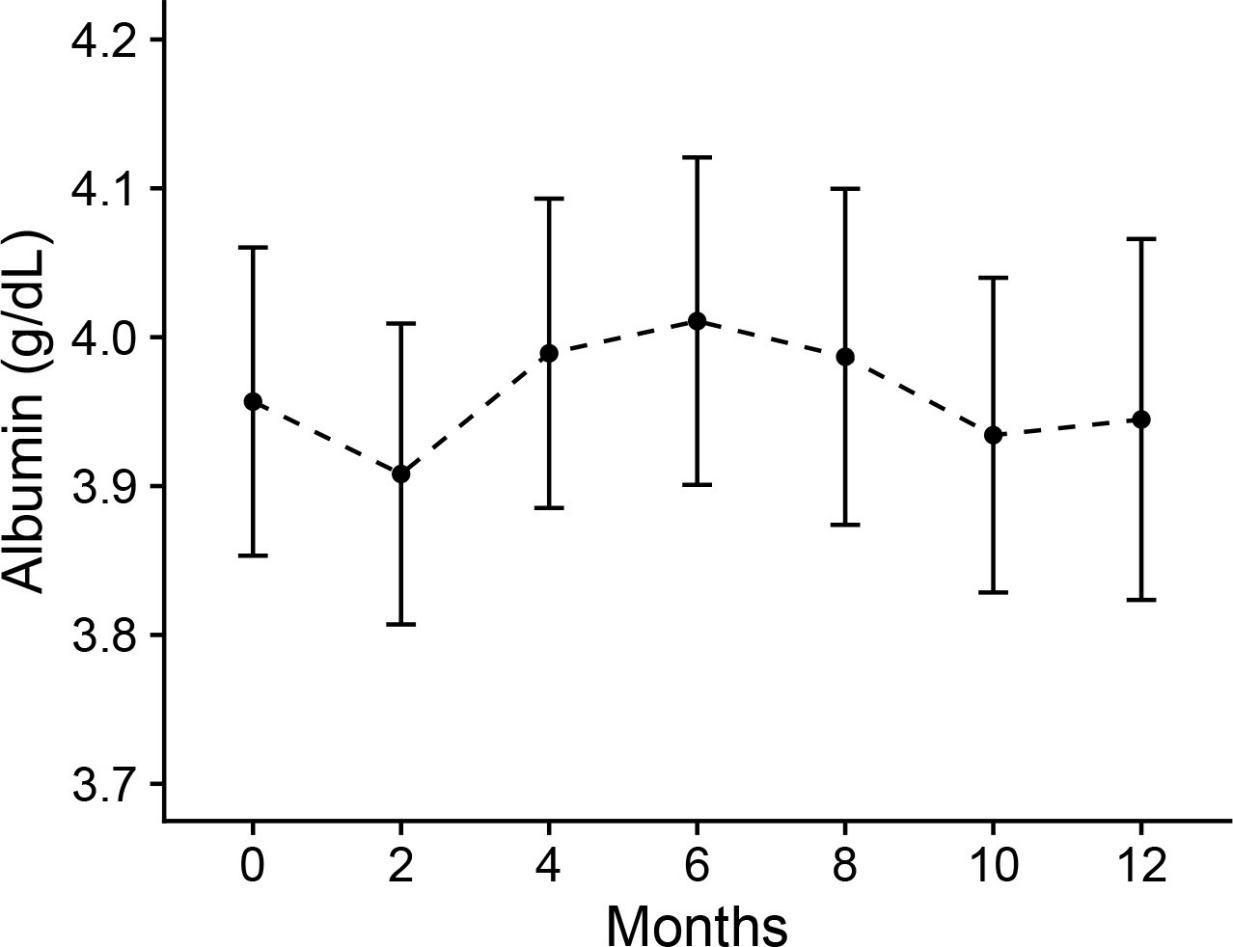
Serum albumin concentration during 1-year treatment with medium cut-off dialyzer. Data are presented as geometric means and 95% confidence intervals (CIs) as error bars.

The levels of basic laboratory parameters and middle molecules before and after the one year treatment are listed in Table 2. In high-flux HD patients, there was no significant changes in listed parameters between baseline and 12 months. After using the MCO dialyzer for one year, serum sodium and potassium concentrations were decreased (sodium, P = 0.006; potassium, P = 0.003), and serum ferritin and β2-microglobulin concentrations were increased (ferritin, P = 0.007; β2-microglobulin, P = 0.046). Large middle molecules, such as lambda and kappa FLC maintained similar concentrations. Serum albumin levels were not significantly decreased over one year of treatment with the MCO dialyzer (Fig 1). In all parameters, the amounts of 1-year changes were not significantly different between the high-flux and MCO groups (S1 Table). Incidence of adverse events were not differ between high-flux group and MCO group (S2 Table). Monthly dosage of ESA was not significantly changed after one-year treatment with MCO dialyzer (S1 Fig). When analyzed with a linear mixed model after the adjustment of baseline ESA doses, there was no difference of ESA doses between high-flux group and MCO group over one-year treatment (P = 0.796).

**Table 2:** Laboratory parameters and levels of middle molecules before and after 12 month treatment with high-flux and medium cut-off dialyzers.

	High-flux HD			MCO HD		
	Baseline	12 months	*P*-value	Baseline	12 months	*P*-value
**Laboratory parameters**						
Hemoglobin, g/dL	10.6 ± 1.5	10.3 ± 1.4	0.437	10.9 ± 1.3	10.6 ± 1.1	0.314
Platelet, ×10^6^/μL	155.1 ± 53.1	150.1 ± 42.5	0.476	181.0 ± 57.0	182.7 ± 59.3	0.805
Total protein, g/dL	7.05 ± 0.48	7.11 ± 0.50	0.436	6.96 ± 0.49	6.96 ± 0.52	0.967
Albumin, g/dL	4.16 ± 0.29	4.16 ± 0.30	0.932	3.96 ± 0.31	3.94 ± 0.37	0.644
Urea nitrogen, mg/dL	62.0 ± 22.6	59.6 ± 14.6	0.684	53.5 ± 16.7	54.7 ± 16.0	0.670
Creatinine, mg/dL	10.2 ± 3.5	10.5 ± 3.4	0.425	9.7 ± 2.9	10.0 ± 2.7	0.323
Total calcium, mg/dL	9.12 ± 0.71	9.15 ± 0.70	0.781	9.17 ± 0.54	9.01 ± 0.68	0.085
Phosphorus, mg/dL	4.96 ± 1.82	4.65 ± 1.79	0.372	4.43 ± 1.47	4.46 ± 1.74	0.847
Sodium, mmol/L	140.1 ± 1.8	138.8 ± 3.7	0.204	139.7 ± 2.2	138.4 ± 2.6	0.006
Potassium, mmol/L	4.92 ± 0.58	4.62 ± 0.75	0.106	5.07 ± 0.78	4.73 ± 0.63	0.003
Ferritin, ng/mL	283 (159–562)	256 (165–529)	0.891	176 (112–388)	338 (190–555)	0.007
Transferrin saturation, %	30.9 (23.9–36.9)	36.2 (29.3–42.8)	0.235	26.7 (21.7–33.8)	31.9 (26.8–45.4)	0.079
**Middle molecules**						
Lambda FLC, mg/L	154.0 ± 72.3	165.9 ± 67.3	0.357	135.7 ± 39.9	132.0 ± 39.1	0.543
Kappa FLC, mg/L	193.3 ± 88.6	199.8 ± 79.5	0.666	168.2 ± 58.5	167.7 ± 65.8	0.904
β2-microglobulin, mg/L	27.6 ± 11.3	28.6 ± 7.4	0.682	25.6 ± 9.6	28.4 ± 4.8	0.046
Vitamin B12, pg/mL	1127.4 ± 339.4	1148.5 ± 297.2	0.792	1128.5 ± 366.6	1118.2 ± 314.9	0.890

Data are presented as mean ± SD and median (interquartile range), as appropriate. *P*-values for difference between baseline and 12 months were calculated by paired samples t-test or Wilcoxon signed-rank test, according to the distribution of variables. MCO, medium cut-off; HD, hemodialysis; FLC, free light chain.

### Solute removal during a hemodialysis session

**Fig 2:**
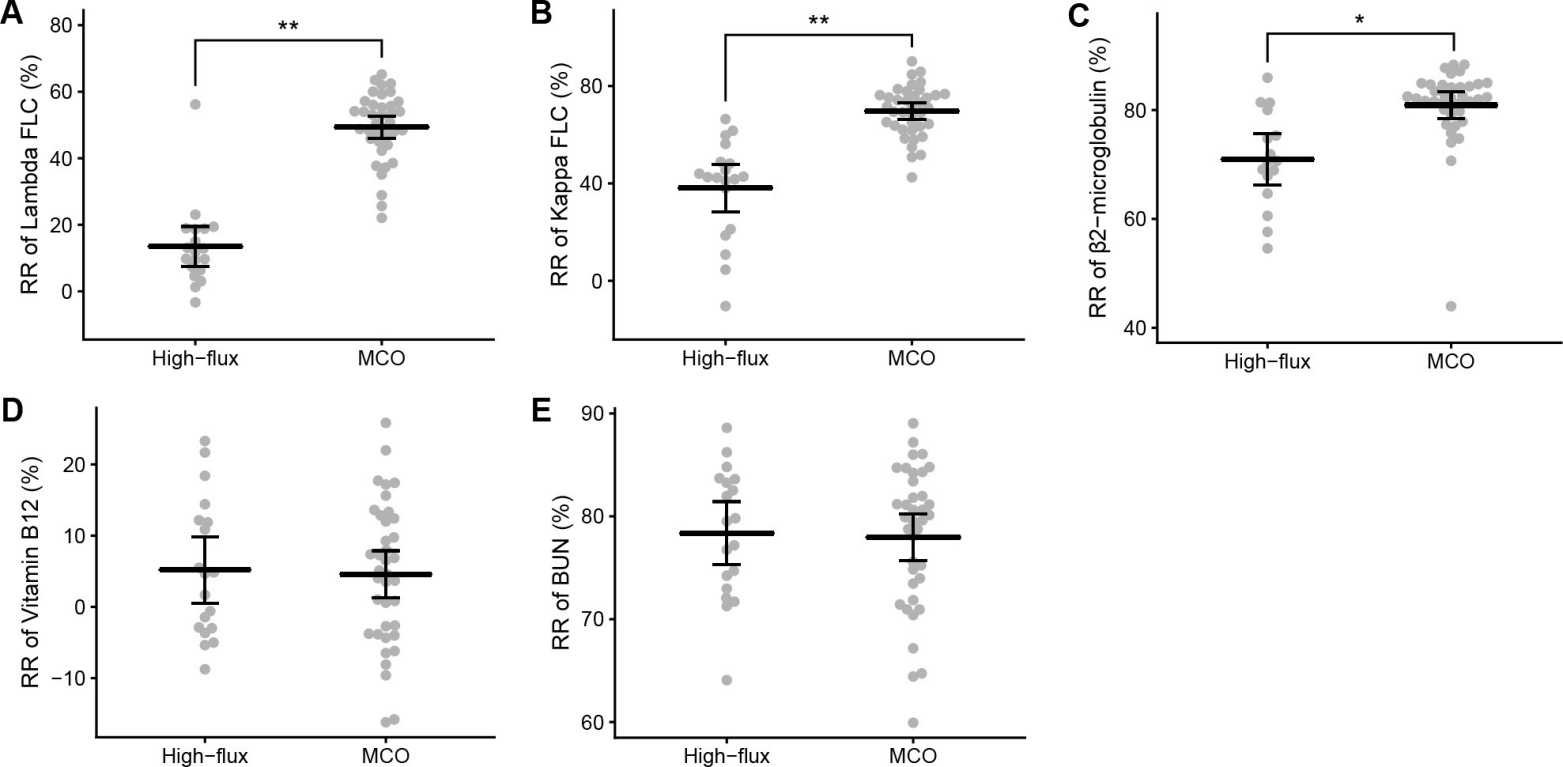
The reduction ratio of small and middle molecules during a hemodialysis session with high-flux and medium cut-off dialyzers. Small and middle molecules, including lambda free light chain (A), kappa free light chain (B), β2-microglobulin (C), Vitamin B12 (D), and urea nitrogen (E) were measured before and after a hemodialysis session. Reduction ratios of middle molecules were calculated after correcting post-dialysis concentrations for hemoconcentration. *P < 0.01; **P < 0.001. MCO, medium cut-off; RR, reduction ratio; FLC, free light chain; BUN, blood urea nitrogen.

Blood level changes of small and middle molecules in each treatment are shown in Table 3. In high-flux HD, serum levels of kappa FLC, β2-microglobulin, vitamin B12, and urea nitrogen were decreased (*P* < 0.001), but lambda FLC was not significantly decreased (*P* = 0.557). In MCO HD, lambda FLC, as well as kappa FLC, β2-microglobulin, vitamin B12, and urea nitrogen were significantly decreased after an HD session (*P* < 0.001). RR of the molecules according to dialyzer types are presented in Fig 2. The RR of middle molecules were adjusted for hemoconcentration. The RR of lambda FLC (high-flux, 13.5 ± 12.5%; MCO, 49.3 ± 10.3%; *P* < 0.001), kappa FLC (high-flux, 38.1 ± 20.3%; MCO, 69.6 ± 10.4%; *P* < 0.001) and β2-microglobulin (high-flux, 71.0 ± 8.8%; MCO, 80.9 ± 7.3%; *P* = 0.002) in MCO HD were higher than those in high-flux HD. The RR of vitamin B12 (high-flux, 5.2 ± 9.7%; MCO, 4.6 ± 10.0%; *P* = 0.830) and urea nitrogen (high-flux, 78.4 ± 6.3%; MCO, 78.0 ± 6.9%; *P* = 0.827) were similar between both dialyzer types.

**Table 3:** Blood level changes in small and middle molecules during a hemodialysis session.

	High-flux HD			MCO HD		
	Pre-HD	Post-HD	*P*-value	Pre-HD	Post-HD	*P*-value
Lambda FLC, mg/L	165.9 ± 67.3	168.3 ± 73.8	0.557	132.0 ± 39.1	83.7 ± 31.6	<0.001
Kappa FLC, mg/L	199.8 ± 79.5	138.4 ± 47.8	<0.001	167.7 ± 65.8	63.0 ± 30.3	<0.001
β2-microglobulin, mg/L	28.6 ± 7.4	10.0 ± 3.9	<0.001	28.4 ± 4.8	6.8 ± 2.9	<0.001
Vitamin B12, pg/mL	1148.5 ± 297.2	1282.3 ± 330.8	<0.001	1118.2 ± 314.9	1301.6 ± 304.5	<0.001
Urea nitrogen, mg/dL	59.6 ± 14.6	12.8 ± 4.6	<0.001	54.7 ± 16.0	12.2 ± 5.8	<0.001

Data are presented as mean ± SD. *P*-values for difference between pre-HD and post-HD blood levels were calculated by paired samples t-test or Wilcoxon signed-rank test. MCO, medium cut-off; HD, hemodialysis; FLC, free light chain.

### Removal of cell-free hemoglobin

**Fig 3:**
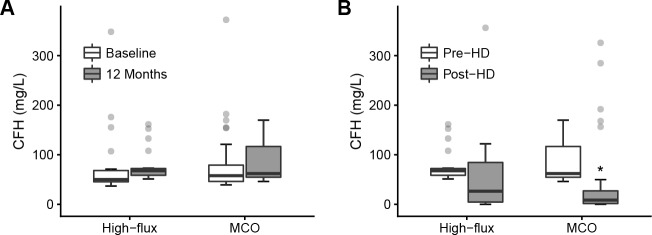
The changes of cell-free hemoglobin concentration with high-flux and medium cut-off dialyzers. (A) The cell-free hemoglobin concentrations before and after 12-month treatment with high-flux and medium cut-off dialyzer are presented. (B) The pre- and post-hemodialysis cell-free hemoglobin concentrations during a hemodialysis session in the high-flux group and medium cut-off group are presented. *P < 0.001. MCO, medium cut-off; CFH, cell-free hemoglobin.

There was no significant difference in serum CFH concentration from baseline to 12 months in either the high-flux group (from 50.0 [45.4–68.1] to 67.7 [58.6–72.3] mg/L, *P* = 0.113) or the MCO group (from 57.8 [46.2–79.1] to 62.0 [54.6–116.7] mg/L, *P* = 0.129) (Fig 3A). During an HD session, median post-HD CFH was lower than median pre-HD CFH in the high-flux group, but it was not statistically significant (from 67.7 [58.6–72.3] to 26.5 [4.6–84.5] mg/L, *P* = 0.240) (Fig 3B). On the other hand, CFH concentrations were significantly decreased during an HD session in the MCO group (from 62.0 [54.6–116.7] to 8.5 [1.6–27.2] mg/L, *P* < 0.001). The RR of CPH was not significantly different between the high-flux HD (68.7 [-6.8–93.6] %) and the MCO HD (85.5 [78.7–97.3] %, *P* = 0.290).

## Discussion

Our clinical study examined long-term effects of the MCO dialyzer compared to the high-flux dialyzer. We showed that reduction ratios of middle and large middle molecules in the MCO group were higher than those of the high-flux group. However, despite a 12-month treatment with the MCO dialyzer, the serum levels of these molecules were not lowered significantly.

The MCO membrane was developed to expand toxin removal while preventing serum albumin leakage, by increasing pore size and reducing pore size distribution compared to high-flux membranes [9]. Recent studies reported that MCO HD removed large middle molecules, including lambda FLC (molecular weight of 45 kDa) and α1-glycoprotein (molecular weight of 41 kDa) more sufficiently than high-flux HD and ol-HDF [10–12, 25]. To evaluate its long-term effect, Zickler et al. conducted a study of the MCO dialyzer, and reported that lambda FLC was significantly reduced after 12 weeks of use [25]. In our study, removal of large middle molecules was excellent during a single MCO HD session, in agreement with other studies, but long-term decreases in serum concentrations of these molecules were not observed. These results indicate that reduction of blood levels of uremic toxins by MCO HD was insufficient in a conventional clinical setting, such as in a thrice weekly HD schedule. Ward et al. described the insufficient long-term β2-microglobulin reduction despite of high clearance rate in hemodiafiltration, because of the rebound of the molecules after hemodialysis session [26]. Limited treatment time may be not enough to remove the toxin reside in tissues and the toxin produced between each hemodialysis session. However, our study showed that MCO membrane’s solute removal capacity might reduce the absolute exposure of uremic toxins over the course of a week. It is unclear whether a reduction in absolute exposure of uremic toxins could improve clinical outcomes. More evidence is required to verify the clinical benefit of MCO dialyzer.

Kirsch et al. reported that the albumin loss in MCO HD was 2.9 (1.5–3.9) g or 3.2 (1.9–3.9) g per session, which was within the range observed in ol-HDF, but higher than in high-flux HD [10, 27]. Another study reported that after a 12-week treatment with the MCO dialyzer, serum albumin levels decreased from 3.72 to 3.64 g/dL (P < 0.0001) [25]. Contrary to this result, there was no significant decrease in serum albumin level during the 12-month observation period in our study. Our results suggest that the MCO dialyzer can be used safely in terms of albumin loss. We believe that albumin loss during an MCO HD session could activate albumin synthesis in the liver to keep serum albumin levels constant. Loss of newly-generated albumin might be beneficial if protein-bound toxins are collaterally cleared.

The CFH concentration in the general population is known to be in the range of 6 and 34 mg/L [16]. Because CFH can be released from destruction of red blood cells during extracorporeal therapies, it is significantly elevated in hemodialysis patients. One study reported that pre-HD CPH concentrations were 196 ± 43 mg/L and increased to 285 ± 109 mg/L after HD with a low-flux dialyzer [15]. The mean CFH concentration in our study was 55.4 [46.1–77.9] mg/L, which was higher than the general population, but lower than previously reported in HD patients. The lower CFH concentrations found in our study compared to the previous study [15] might be caused by improved biocompatibility of the membrane and relatively lighter participants (58 kg versus 74 kg). In our data, CFH was significantly correlated with dry weight of participants (*R* = 0.395, *P* = 0.002). Because the previous study, which was conducted with bovine blood, reported that CFH could be removed during MCO HD, we focused on CFH removal in HD patients using the MCO dialyzer. In our study, CFH was significantly reduced during an HD session with the MCO dialyzer, but not with the high-flux dialyzer. Nonetheless, as with large middle molecules, CFH decreased by MCO HD was not found during one year of follow-up. The RR of CFH was higher than expected, given the high molecular weight of 62.6 kDa. CFH can be dissociated from a tetramer into dimers with molecular weights of 31.3 kDa, with varying degrees of dissociation [21]. The beneficial effect of CFH removal during a HD session has not been investigated.

There were a few limitations to this study. First, we conducted an observational cohort study, instead of a randomized controlled trial, which could lead to selection bias. Second, evaluation of clinical outcomes was limited in this study. The information about adverse events and ESA dosage have been provided in this study, but these results have to be carefully interpreted because of the limited number of subjects and just one year follow up period. Other clinical endpoints, such as quality of life or nutritional status, need to be studied in future research. Our study also could not determine whether removal of CFH during an HD session reduced CFH-related toxicity. Third, ol-HDF data was not included in this study. We could not include ol-HDF participants because ol-HDF has been assigned to symptomatic or imbalanced patients in our dialysis unit. More comparison data are required because previous studies showed conflicting results in the comparison of solute removal between ol-HDF and MCO HD [10, 12]. Fourth, residual renal functions of the participants were not accurately investigated because the amount of urine output and 24-hour urine data was not collected in this study. Instead, residual renal functions were estimated by baseline β2-microglobulin levels using two different estimating equations [22, 23], and there were no significant differences between two groups. Residual renal function is very important because it could influence the long-term clearance of middle molecules. Although the dialysis vintage tended to be a little shorter than the high-flux group in the MCO group (dialysis vintage of high-flux HD group, 120.0 [28.0–192.5] months; MCO group, 71.5 [45.0–151.3] months), there was no statistical significance (P = 0.767). This trend could not be ignored because the limited number of participants could make the tests insignificant. We need to be careful in interpreting the results because of the possible differences of residual renal function originated from the possible difference of dialysis vintage between two groups.

In conclusion, there was no significant long-term reduction of large middle molecules and CFH during 1-year treatment with the MCO dialyzer. The clearance of large middle molecules was much improved in the MCO dialyzer group, compared to the high-flux dialyzer group, without long-term albumin loss. The MCO dialyzer can be used effectively and safely in conventional HD settings. Future studies are warranted to reveal the clinical impact of the MCO dialyzer, especially on aspects of mortality and cardiovascular events.

## Supporting information

S1 Table Changes in laboratory parameters and middle molecules over a 12-month treatment with high-flux and medium cut-off dialyzers. (DOCX)

S2 Table Incidence of adverse events over a 12-month treatment with high-flux and medium cut-off dialyzers. (DOCX)

S1 Fig Monthly changes of darbepoietin-alpha dosage over a 12-month treatment with high-flux and medium cut-off dialyzers. Data are presented as geometric means and 95% confidence intervals as error bars.
(TIF)

## References

[1] Vanholder R, Massy Z, Argiles A, Spasovski G, Verbeke F, Lameire N. Chronic kidney disease as cause of cardiovascular morbidity and mortality. Nephrol Dial Transplant. 2005;20(6):1048–56. 10.1093/ndt/gfh813 15814534

[2] Matsushita K, van der Velde M, Astor BC, Woodward M, Levey AS, de Jong PE, et al Association of estimated glomerular filtration rate and albuminuria with all-cause and cardiovascular mortality in general population cohorts: a collaborative meta-analysis. Lancet. 2010;375(9731):2073–81. 10.1016/S0140-6736(10)60674-5 20483451 PMC3993088

[3] Vanholder R, Baurmeister U, Brunet P, Cohen G, Glorieux G, Jankowski J. A Bench to Bedside View of Uremic Toxins. J Am Soc Nephrol. 2008;19(5):863–70. 10.1681/ASN.2007121377 18287557

[4] Vanholder R, De Smet R, Glorieux G, Argilés A, Baurmeister U, Brunet P, et al Review on uremic toxins: Classification, concentration, and interindividual variability. Kidney Int. 2003;63(5):1934–43. 10.1046/j.1523-1755.2003.00924.x 12675874

[5] Cheung AK, Rocco MV, Yan G, Leypoldt JK, Levin NW, Greene T, et al Serum β-2 Microglobulin Levels Predict Mortality in Dialysis Patients: Results of the HEMO Study. J Am Soc Nephrol. 2006;17(2):546–55. 10.1681/ASN.2005020132 16382021

[6] Desjardins L, Liabeuf S, Lenglet A, Lemke HD, Vanholder R, Choukroun G, et al Association between free light chain levels, and disease progression and mortality in chronic kidney disease. Toxins (Basel). 2013;5(11):2058–73. 10.3390/toxins5112058 24217396 PMC3847714

[7] Cohen G, Haag-Weber M, Mai B, Deicher R, Horl WH. Effect of immunoglobulin light chains from hemodialysis and continuous ambulatory peritoneal dialysis patients on polymorphonuclear leukocyte functions. J Am Soc Nephrol. 1995;6(6):1592–9. 8749685 10.1681/ASN.V661592

[8] Lamy T, Henri P, Lobbedez T, Comby E, Ryckelynck JP, Ficheux M. Comparison between on-line high-efficiency hemodiafiltration and conventional high-flux hemodialysis for polyclonal free light chain removal. Blood Purif. 2014;37(2):93–8. 10.1159/000357968 24603634

[9] Boschetti-de-Fierro A, Voigt M, Storr M, Krause B. MCO Membranes: Enhanced Selectivity in High-Flux Class. Sci Rep. 2015;5:18448 10.1038/srep18448 26669756 PMC4680880

[10] Kirsch AH, Lyko R, Nilsson LG, Beck W, Amdahl M, Lechner P, et al Performance of hemodialysis with novel medium cut-off dialyzers. Nephrol Dial Transplant. 2017;32(1):165–72. 10.1093/ndt/gfw310 27587605 PMC5837492

[11] Kirsch AH, Rosenkranz AR, Lyko R, Krieter DH. Effects of Hemodialysis Therapy Using Dialyzers with Medium Cut-Off Membranes on Middle Molecules. Contrib Nephrol. 2017;191:158–67. 10.1159/000479264 28910799

[12] García-Prieto A, Vega A, Linares T, Abad S, Macías N, Aragoncillo I, et al Evaluation of the efficacy of a medium cut-off dialyser and comparison with other high-flux dialysers in conventional haemodialysis and online haemodiafiltration. Clin Kidney J. 2018;11(5):742–6. 10.1093/ckj/sfy004 30288272 PMC6165747

[13] Polaschegg HD. Red blood cell damage from extracorporeal circulation in hemodialysis. Semin Dial. 2009;22(5):524–31. 10.1111/j.1525-139X.2009.00616.x 19558629

[14] Sakota R, Lodi CA, Sconziano SA, Beck W, Bosch JP. In Vitro Comparative Assessment of Mechanical Blood Damage Induced by Different Hemodialysis Treatments. Artif Organs. 2015;39(12):1015–23. 10.1111/aor.12499 25981394 PMC5029586

[15] Meyer C, Heiss C, Drexhage C, Kehmeier ES, Balzer J, Muhlfeld A, et al Hemodialysis-induced release of hemoglobin limits nitric oxide bioavailability and impairs vascular function. J Am Coll Cardiol. 2010;55(5):454–9. 10.1016/j.jacc.2009.07.068 20117459

[16] Fairbanks VF, Ziesmer SC, O'Brien PC. Methods for measuring plasma hemoglobin in micromolar concentration compared. Clin Chem. 1992;38(1):132–40. 1733585

[17] Reiter CD, Wang X, Tanus-Santos JE, Hogg N, Cannon RO 3rd, Schechter AN, et al Cell-free hemoglobin limits nitric oxide bioavailability in sickle-cell disease. Nat Med. 2002;8(12):1383–9. 10.1038/nm799 12426562

[18] Yeo TW, Lampah DA, Tjitra E, Gitawati R, Kenangalem E, Piera K, et al Relationship of cell-free hemoglobin to impaired endothelial nitric oxide bioavailability and perfusion in severe falciparum malaria. J Infect Dis. 2009;200(10):1522–9. 10.1086/644641 19803726 PMC3740798

[19] Adamzik M, Hamburger T, Petrat F, Peters J, de Groot H, Hartmann M. Free hemoglobin concentration in severe sepsis: methods of measurement and prediction of outcome. Crit Care. 2012;16(4):R125 10.1186/cc11425 22800762 PMC3580706

[20] Baek JH, D’Agnillo F, Vallelian F, Pereira CP, Williams MC, Jia Y, et al Hemoglobin-driven pathophysiology is an in vivo consequence of the red blood cell storage lesion that can be attenuated in guinea pigs by haptoglobin therapy. J Clin Invest. 2012;122(4):1444–58. 10.1172/JCI59770 22446185 PMC3314461

[21] Hulko M, Kunz M, Yildirim M, Homeyer S, Amon O, Krause B. Cell-free plasma hemoglobin removal by dialyzers with various permeability profiles. Sci Rep. 2015;5:16367 10.1038/srep16367 26553708 PMC4639840

[22] Shafi T, Michels WM, Levey AS, Inker LA, Dekker FW, Krediet RT, et al Estimating residual kidney function in dialysis patients without urine collection. Kidney Int. 2016;89(5):1099–110. 10.1016/j.kint.2015.10.011 26924062 PMC4834223

[23] Vilar E, Boltiador C, Wong J, Viljoen A, Machado A, Uthayakumar A, et al Plasma Levels of Middle Molecules to Estimate Residual Kidney Function in Haemodialysis without Urine Collection. PLoS One. 2015;10(12):e0143813 10.1371/journal.pone.0143813 26629900 PMC4668015

[24] Bergstrom J, Wehle B. No change in corrected beta 2-microglobulin concentration after cuprophane haemodialysis. Lancet. 1987;1(8533):628–9. 10.1016/s0140-6736(87)90266-2 2881162

[25] Zickler D, Schindler R, Willy K, Martus P, Pawlak M, Storr M, et al Medium Cut-Off (MCO) Membranes Reduce Inflammation in Chronic Dialysis Patients—A Randomized Controlled Clinical Trial. PLoS ONE. 2017;12(1):e0169024 10.1371/journal.pone.0169024 28085888 PMC5234772

[26] Ward RA, Greene T, Hartmann B, Samtleben W. Resistance to intercompartmental mass transfer limits beta2-microglobulin removal by post-dilution hemodiafiltration. Kidney Int. 2006;69(8):1431–7. 10.1038/sj.ki.5000048 16395268

[27] Meert N, Eloot S, Schepers E, Lemke HD, Dhondt A, Glorieux G, et al Comparison of removal capacity of two consecutive generations of high-flux dialysers during different treatment modalities. Nephrol Dial Transplant. 2011;26(8):2624–30. 10.1093/ndt/gfq803 21310741

