# Supplementary material for: Long-term effect of medium cut-off dialyzer on middle uremic toxins and cell-free hemoglobin
Source: PLoS One. 2019 Jul 26;14(7):e0220448. doi: 10.1371/journal.pone.0220448 (PMC6660073; doi:10.1371/journal.pone.0220448)
Supplement: S1 Table — (DOCX) [file pone.0220448.s001.docx]

**S1 Table. Changes in laboratory parameters and middle molecules over a 12-month treatment with high-flux and medium cut-off dialyzers.**

|  | Changes (Δ) between baseline and 12 months | | |
| --- | --- | --- | --- |
|  | High-flux HD | MCO HD | *P*-value |
| **Laboratory parameters** |  |  |  |
| Hemoglobin, g/dL | -0.33 + 1.79 | -0.26 + 1.57 | 0.893 |
| Platelet, ×10^6^/μL | -5.00 + 29.95 | 1.74 + 43.15 | 0.495 |
| Total protein, g/dL | 0.06 + 0.32 | 0.00 + 0.40 | 0.537 |
| Albumin, g/dL | 0.01 + 0.26 | -0.02 + 0.28 | 0.726 |
| Urea nitrogen, mg/dL | -6.54 + 23.72 | 1.17 + 16.80 | 0.216 |
| Creatinine, mg/dL | 0.31 + 1.67 | 0.31 + 1.91 | 0.998 |
| Total calcium, mg/dL | 0.03 + 0.49 | -0.18 + 0.60 | 0.173 |
| Phosphorus, mg/dL | -0.32 + 1.50 | 0.06 + 1.78 | 0.415 |
| Sodium, mmol/L | -1.26 + 4.17 | -1.34 + 2.82 | 0.941 |
| Potassium, mmol/L | -0.30 + 0.77 | -0.34 + 0.65 | 0.859 |
| Ferritin, ng/mL | 16.5 (-142.4, 97.9) | 63.0 (-13.0, 249.5) | 0.083 |
| Transferrin saturation, % | 2.2 (-4.4, 16.6) | 9.3 (-5.0, 18.5) | 0.678 |
| **Middle molecules** |  |  |  |
| Lambda FLC, mg/L | 11.9 + 54.8 | -2.7 + 26.2 | 0.286 |
| Kappa FLC, mg/L | 6.5 + 64.6 | 1.1 + 53.3 | 0.754 |
| β2-microglobulin, mg/L | -0.74 + 7.11 | 2.83 + 8.47 | 0.121 |
| Vitamin B12, pg/mL | 21.1 + 342.7 | -5.8 + 252.7 | 0.765 |

Data are presented as mean ± SD, or median (interquartile range), as appropriate. Changes (Δ) were calculated by subtracting the baseline values from the 12 month values. *P*-values were calculated by Student’s t-test for normally distributed continuous variables, and by Mann–Whitney U test for non-normally distributed continuous variables. MCO, medium cut-off; HD, hemodialysis; FLC, free light chain.
