# Supplementary material for: Long-term effect of medium cut-off dialyzer on middle uremic toxins and cell-free hemoglobin
Source: PLoS One. 2019 Jul 26;14(7):e0220448. doi: 10.1371/journal.pone.0220448 (PMC6660073; doi:10.1371/journal.pone.0220448)
Supplement: S2 Table — (DOCX) [file pone.0220448.s002.docx]

**S2 Table. Incidence of adverse events over a 12-month treatment with high-flux and medium cut-off dialyzers.**

| Adverse events | High-flux HD | MCO HD | *P*-value |
| --- | --- | --- | --- |
| Mortality, *n* (%) | 0 (0) | 0 (0) | 1.000 |
| Cardiovascular events, *n* (%) | 1 (5.3)^a^ | 0 (0) | 0.333 |
| Infections, *n* (%) | 2 (10.5)^b^ | 5 (13.2)^c^ | 1.000 |

^a^Cardiovascular event in high-flux HD group was nonfatal stroke. ^b^Infections in high-flux HD group were two infectious colitis. ^c^Infections in MCO HD group were cellulitis, acute mastoiditis, and three infectious colitis. *P*-values were calculated by Fisher’s exact test. MCO, medium cut-off; HD, hemodialysis.
