# Supplementary figures and images for: Long-term effect of medium cut-off dialyzer on middle uremic toxins and cell-free hemoglobin
Source: PLoS One. 2019 Jul 26;14(7):e0220448. doi: 10.1371/journal.pone.0220448 (PMC6660073; doi:10.1371/journal.pone.0220448)

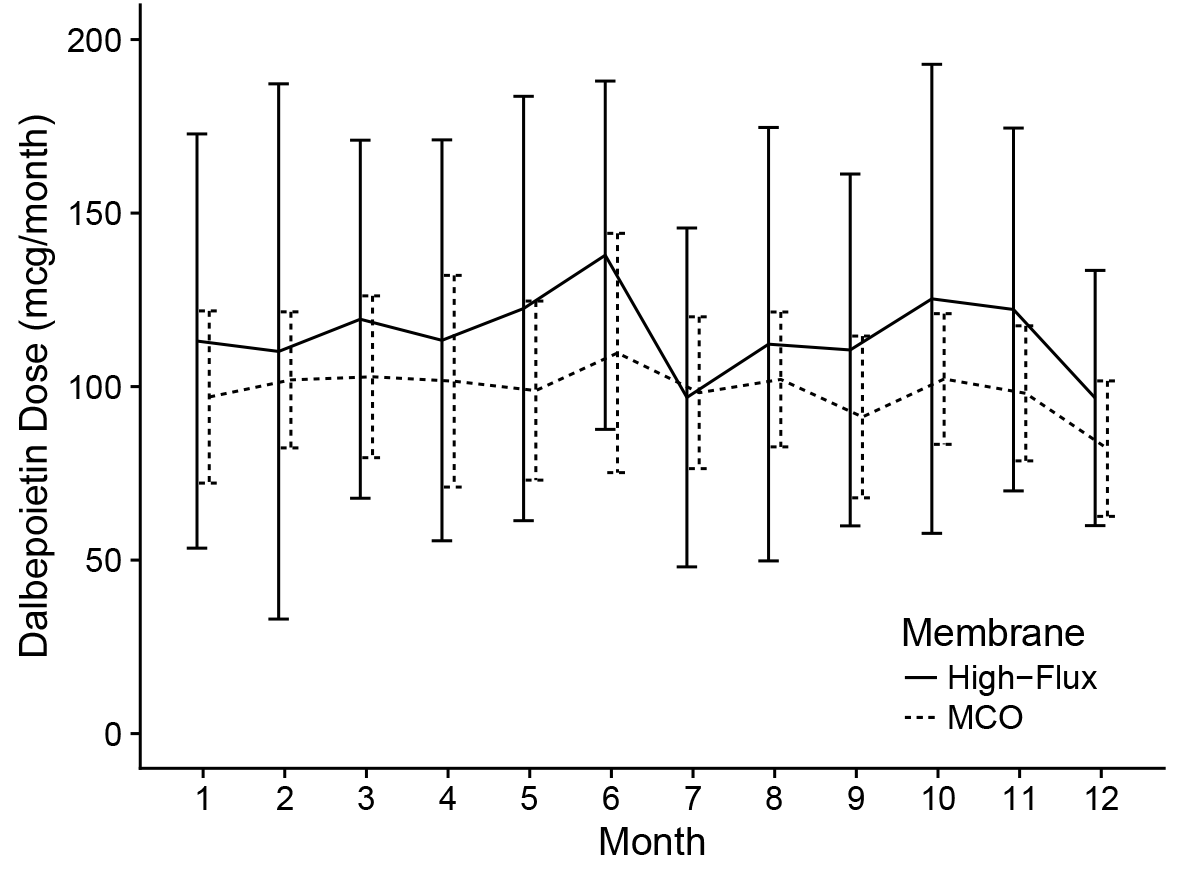

Supplement: S1 Fig — Data are presented as geometric means and 95% confidence intervals as error bars. (TIF) [file pone.0220448.s003.tif]
